# Probable pyoderma gangrenosum complicated with cutaneous *Aspergillus flavus* infection: case report

**DOI:** 10.3389/fmed.2026.1806433

**Published:** 2026-04-22

**Authors:** Shaoqin Sun, Mengting Zhu, Changhui Wen

**Affiliations:** 1 Department of Dermatology, First Affiliated Hospital of Guizhou University of Traditional Chinese Medicine, Guiyang, Guizhou, China; 2 Guizhou University of Traditional Chinese Medicine, Guiyang, Guizhou, China

**Keywords:** *Aspergillus flavus*, cutaneous aspergillosis, negative pressure wound therapy, pyoderma gangrenosum, voriconazole

## Abstract

Pyoderma gangrenosum (PG) is a rare, chronic, autoinflammatory neutrophilic dermatosis. Its typical clinical manifestations include painful papules and pustules surrounded by erythema on the lower extremities and trunk, which gradually progress to form well-demarcated, painful ulcers with undermined edges. Due to impaired skin barrier function, prolonged wound exposure, and the frequent need for immunosuppressive therapy, PG patients are highly susceptible to secondary infections. We report here a clinical case of successful treatment with glucocorticoids and voriconazole, combined with multiple sessions of negative-pressure wound therapy and skin grafting, in a patient with cutaneous *Aspergillus flavus* infection and possible pyoderma gangrenosum.

## Case presentation

We present the case of a 69-year-old man who had papules and ulcers on the trunk and extremities, accompanied by pruritus and pain for over a year, which worsened in the past month.

More than a year ago, the patient developed papules on the trunk and extremities without an obvious inducement, accompanied by pruritus and pain. Repeated scratching led to the formation of localized ulcers covered with crusts, which progressively expanded. The skin lesions showed no improvement despite self-administration of topical ointments and treatment at a local clinic. Details of the specific diagnosis and treatment plan were unavailable.

More than 3 months before presentation, the patient was admitted to our department for recurrence of papules and ulcers on the trunk and extremities, accompanied by pruritus and pain for over a year. On admission, skin examination revealed numerous scattered, round-like ulcers on the neck, shoulders, lumbogluteal region, and extremities, some of which were fused into sheets and were covered with crusts. The lesion areas were dry, with no purulent secretions. After admission, a series of examinations were completed, including routine blood test, liver and kidney function tests, urine routine, electrolyte test, infectious markers, stool routine, pemphigus and pemphigoid antibodies, erythrocyte sedimentation rate, antinuclear antibody profile, vasculitis-related tests, complete immune function tests, and digital chest radiography, all of which were negative. A skin biopsy was performed on the skin tissue of the right upper arm, and the pathological report indicated a chronic skin ulcer with dense inflammatory cell infiltration surrounding the small blood vessels in the dermis. Acid-fast staining and wound secretion cultures were negative. Given that the ulcer had no obvious cause and was a non-venous, non-infectious chronic ulcer, its features were consistent with the precursor ulcer of pyoderma gangrenosum, although the lesion had not progressed to a typical deep ulcer with undermined edges. Therefore, a provisional diagnosis of chronic skin ulcer was made. The patient received comprehensive treatments, including dexamethasone sodium phosphate injection for anti-inflammation, vitamin C injection, and calcium gluconate injection, to reduce capillary permeability, along with antihistamine, antipruritic, and analgesic therapies, after which the ulcers improved, and he was discharged from the hospital. At discharge, the skin condition was as follows: most of the crusts on the ulcers of the neck, shoulders, lumbogluteal region, and extremities had fallen off, and fresh granulation tissue and epithelial islands were visible on multiple ulcer surfaces. After discharge, the patient intermittently used topical halometasone cream to control the condition.

In the past month, the skin ulcers worsened without obvious inducement, and three new deep ulcers appeared on the left lower extremity, with progressively enlarging areas and severe pain. Topical self-application of halometasone cream failed to improve the ulcers or related symptoms; therefore, he visited our department again.

The patient had a generally good health status with no underlying diseases. He denied a history of hypertension and diabetes, hepatitis, tuberculosis, or tumors, as well as any history of trauma or surgery. He also denied a history of food and drug allergies. There was no notable family history.

**Figure 1:**
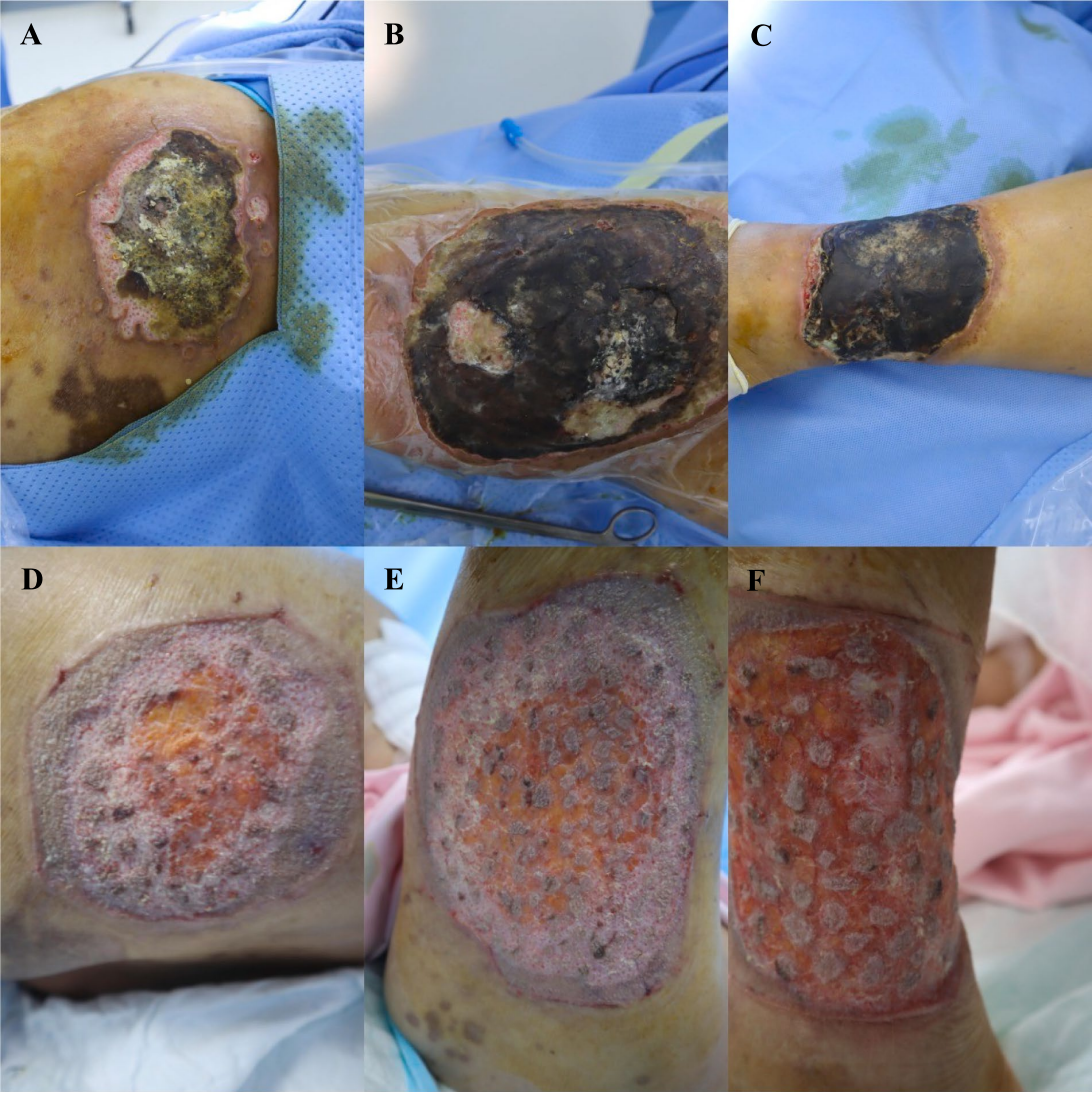
**(A)** Before treatment: skin condition of the patient. The affected area is the left lumbogluteal region, and a deep ulcer with a size of approximately 8.0 × 9.0 cm is observed. The ulcer margins are purplish–red and partially undermined. The ulcers are covered with brown crusts and powdery materials, with sanguinopurulent exudate and yellowish–white secretions present. **(B)** Before treatment: skin condition of the patient. The affected area is the lateral aspect of the left thigh, and a deep ulcer with a size of approximately 15.0*13.0 cm is observed. The ulcer margins are purplish–red and partially undermined. The ulcers are covered with brown crusts and powdery materials, with sanguinopurulent exudate and yellowish–white secretions present. **(C)** Before treatment: skin condition of the patient. The affected area is the lateral aspect of the left lower leg, and a deep ulcer with a size of approximately 10.0 × 8.5 cm is observed. The ulcer margins are purplish–red and partially undermined. The ulcers are covered with brown crusts and powdery materials, with sanguinopurulent exudate and yellowish–white secretions present. **(D)** After four sessions of NPWT and skin grafting: skin condition of the patient. The area in question is the left lumbogluteal region; the wound surface is observed to be clean and dry. Most of the skin grafts have fused into a sheet; partial epithelialization changes are visible, with no oozing blood or purulent secretions attached. **(E)** After four sessions of NPWT and skin grafting: skin condition of the patient. The area in question is the lateral aspect of the left thigh; the wound surface is observed to be clean and dry. Most of the skin grafts have fused into a sheet; partial epithelialization changes are visible, with no oozing blood or purulent secretions attached. **(F)** After four sessions of NPWT and skin grafting: skin condition of the patient. The area in question is the lateral aspect of the left lower leg; the wound surface is observed to be clean and dry. Most of the skin grafts have fused into a sheet; partial epithelialization changes are visible, with no oozing blood or purulent secretions attached.

Physical examination: Body temperature 36.6 °C; respiratory rate 20 breaths per minute; heart rate 94 beats per minute; no obvious abnormalities were noted on systemic examinations. Dermatological examination: Three irregularly shaped deep ulcers were found in the left lumbogluteal region, the lateral aspect of the left thigh, and the lateral aspect of the left calf, with sizes of 8 × 9 cm, 15 × 13 cm, and 10 × 8.5 cm, respectively. The ulcer edges were purplish-red, with undermined areas in some parts, covered with brown crusts and powdery substances, and accompanied by oozing of purulent exudate and adhesion of yellowish-white secretions. Numerous dark brown pigmented spots and scars were visible on the remaining trunk and extremities (Figures 1A–C: pre-treatment skin condition of the patient).

Laboratory examinations showed a white blood cell count of 10.20 × 10^9^/L, a C-reactive protein (CRP) level of 30.20 mg/L, and an erythrocyte sedimentation rate (ESR) of 30.00 mm/h. Liver and kidney function, urine routine, electrolytes, infectious markers, stool routine, coagulation function, antinuclear antibody profile, anti-neutrophil cytoplasmic antibody, complete immune function tests, lupus anticoagulant, and anticardiolipin antibodies were all unremarkable. Chest CT showed the following: 1. chronic bronchitis, pulmonary emphysema, and scattered inflammation in both lungs; 2. multiple small nodules in both lungs; and 3. multiple fibrotic foci in both lungs, with local thickening and adhesion of the bilateral pleura. The tuberculosis purified protein derivative test result was negative. No obvious abnormalities were found on venography of both lower extremities.

On 8 May, the patient underwent the first session of negative pressure wound therapy on the left lower extremity. Intraoperatively, a surgical incision line was designed from the lesion area to the normal skin on the left thigh, and layers were incised down to the fat layer along the line, with partial tissue excised for pathological examination. The crusts and necrotic tissue of the ulcers were debrided one by one, revealing that most of the necrotic tissue extended deep into the deep fascial layer with a large accumulation of thick purulent secretions. Partial deep necrotic tissue was collected for pathological examination, and basal purulent secretions were sampled for wound secretion culture. Postoperatively, analgesic and anti-infective treatments were administered.

**Figure 2:**
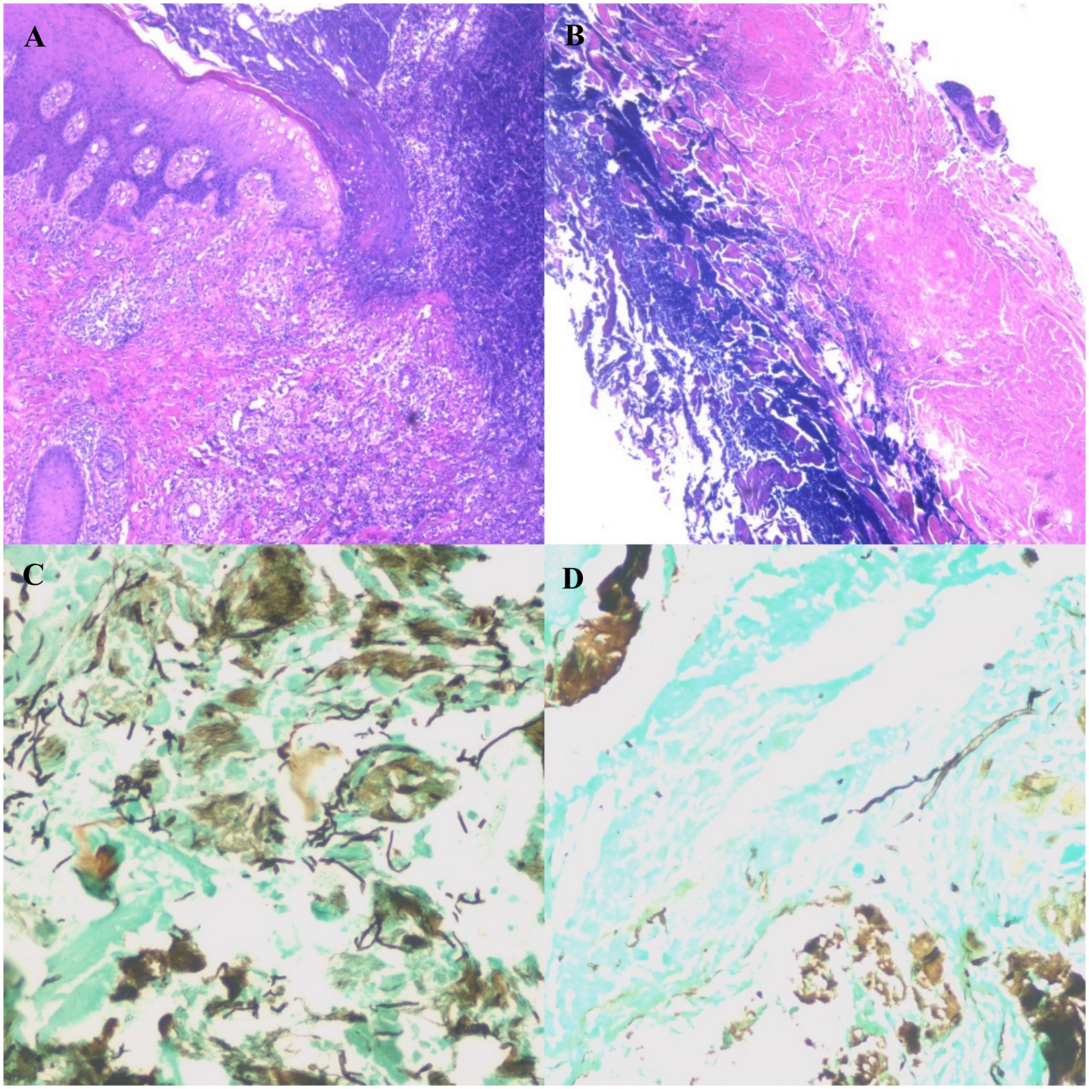
**(A)** Pathological examination result of the skin lesion on the patient’s left lower extremity. The dermis shows edema and loosening, with diffuse inflammatory cell infiltration dominated by neutrophils and mixed with lymphocytes and histiocytes. The blood vessels in the dermis are dilated and congested, and tissue necrosis can be seen in some areas. **(B)** Pathological examination result of the skin lesion on the patient’s left lower extremity. Massive proliferation of fibrous connective tissue is observed, accompanied by dense inflammatory cell infiltration, which is dominated by lymphocytes and histiocytes. Hyperplasia with structural disorder can be seen in some areas. **(C)** Gomori methenamine silver (GMS) staining result of the skin lesion on the patient’s left lower extremity. Blackish-brown hyphae and spores are observed. In some areas, the hyphae are dense and distributed in a reticular pattern. Slender septate hyphae can be seen, with the hyphae branching at a 45° acute angle. **(D)** Gomori methenamine silver (GMS) staining result of the skin lesion on the patient’s left lower extremity. Blackish-brown septate hyphae are observed, with the hyphae branching at a 45° acute angle.

Additional examination results on 14 May: Pathological examination of skin lesion tissue: ① (ulcer tissue of the left lower extremity): chronic skin ulcer complicated by suppurative inflammation and necrosis. The dermis showed edema and loosening, with diffuse inflammatory cell infiltration dominated by neutrophils and mixed with lymphocytes and histiocytes. ② (necrotic tissue of the left lower extremity): numerous deeply stained small blue cells were observed in the fibrous tissue, and the findings, combined with immunohistochemistry, supported the diagnosis of suppurative inflammation with necrosis. A massive proliferation of fibrous connective tissue was observed, accompanied by dense inflammatory cell infiltration dominated by lymphocytes and histiocytes. Immunohistochemical findings: Small blue cells were negative for Cluster of differentiation20, CD3, CD5, CD79a, CD99, Leukocyte common antigen, S-100, CD56, Chromogranin A, desmin, Synaptophysin, vimentin, and Cytokeratin, with equivocal positivity for SMA and a Ki67 proliferation index of <5%. Special staining: Gomori methenamine silver (GMS) staining showed blackish-brown hyphae and spores. Slender septate hyphae were observed, with the hyphae branching at a 45° acute angle (Figures 2A,B: Pathological examination of the skin lesion on the patient’s left lower extremity. Figures 2C,D: Gomori methenamine silver (GMS) staining of the skin lesion on the patient’s left lower extremity). Wound exudate culture was positive for *Aspergillus flavus*. Gram staining and acid-fast staining of wound secretions showed no bacterial growth. Serum 1,3-β-D-glucan: <10.000 pg./mL (reference range: 0–60 pg/mL).

Preliminary diagnosis: (1) possible pyoderma gangrenosum; (2) cutaneous *Aspergillus flavus* infection. Antibiotics were discontinued, and oral prednisone acetate 20 mg/day (for anti-inflammation) and itraconazole capsules (for antifungal therapy) were initiated.

On 16 May, the second session of NPWT was performed. Postoperatively, antifungal therapy was escalated to voriconazole, whereas the dose of prednisone acetate remained unchanged. Four days after surgery, a review showed that the C-reactive protein level had decreased to 2.85 mg/L and the erythrocyte sedimentation rate to 20.00 mm/h. The ulcer size had not increased, with the presence of fine punctate newly formed granulation tissue and petechial hemorrhage in some areas. The pain at the ulcer site was tolerable, indicating that the current dose of glucocorticoid had effectively controlled the inflammation of pyoderma gangrenosum (PG).

On 26 May, the third session of NPWT was conducted, simultaneously with skin harvesting from the right thigh and split-thickness skin grafting (STSG) on the left lower extremity. Intraoperatively, secretions from the ulcer base were collected for wound secretion culture. Five days after skin grafting, following an Aspergillus GM test of <0.50 signal-to-cutoff ratio (S/CO), negative wound secretion culture, no evidence of fungal or bacterial infection, and good skin graft survival, the anti-inflammatory regimen was escalated to intravenous methylprednisolone sodium succinate 40 mg/day, whereas voriconazole was continued for antifungal therapy.

On 6 June, preoperative C-reactive protein and erythrocyte sedimentation rate were both within normal ranges. The patient reported pain at the ulcer site during dressing changes. On examination, the ulcer wound was dry, with pink granulation tissue present, and the skin flap had survived well. The fourth session of NPWT was performed. On the 7th postoperative day, the device was removed, and the wound surfaces in the left lumbogluteal region, the lateral aspect of the left thigh, and the lateral aspect of the left calf measured 6 × 8 cm, 11 × 5 cm, and 9 × 8 cm, respectively. The skin grafts were epithelizing peripherally, with no bleeding or purulent secretions (Figures 1D–F: After four sessions of NPWT and skin grafting, the patient’s skin condition).

The patient was discharged on 25 June and continued oral prednisone acetate 25 mg/day, which was gradually tapered over approximately 1 month until discontinuation. No recurrence was observed during the 3-month follow-up period.

## Diagnosis

Given that pyoderma gangrenosum (PG) remains a presumptive and exclusionary diagnosis (1), it is necessary to outline the patient’s diagnostic process.

First, the clinical course, symptoms, and ulcer characteristics of the patient were consistent with those of the ulcerative variant of pyoderma gangrenosum (PG).

Second, the onset of PG-related skin lesions occurred much earlier than the detection of fungi. The *Aspergillus flavus* infection was considered a recent secondary event, resulting from skin barrier disruption caused by recurrent chronic skin ulcers. The presence of chronic skin ulcers predisposed the patient to colonization and subsequent progression to invasive infection by *Aspergillus flavus*, rather than being the direct cause of the current skin ulcers.

Third, based on the patient’s medical history, symptoms, physical findings, and auxiliary examinations, vasculitis, vascular diseases, malignant tumors, and other autoimmune skin diseases that could cause skin ulcers were excluded.

Fourth, histopathological examination was consistent with active ulcerative PG: predominant neutrophilic infiltration from the dermis to the subcutaneous tissue, accompanied by pathological changes of chronic inflammation consistent with the reparative phase of PG ulcers. No features such as suppurative granulomatous inflammation, fungal infiltration along vascular walls, or coagulative tissue necrosis were observed, indicating that the dominant inflammatory process was the autoimmune inflammation of PG.

Fifth, the ulcer improved after previous anti-inflammatory treatment with dexamethasone sodium phosphate injection. After admission, inflammatory markers decreased following systemic glucocorticoid therapy, and skin lesions and pain were controlled, consistent with the characteristic sensitivity to glucocorticoids. Furthermore, antifungal therapy was effective concurrently after glucocorticoid control of PG inflammation, with no independent evidence of fungal recurrence.

Using the PARACELSUS Score (2019), the final diagnoses were as follows: 1. possible pyoderma gangrenosum and 2. cutaneous *Aspergillus flavus* infection.

## Discussion

Infection with *Aspergillus flavus* (AF) is more prevalent in immunocompromised patients, such as those with diabetes mellitus, individuals on long-term glucocorticoid therapy, and recipients of hematopoietic stem cell transplantation and solid organ transplantation (2). Given the non-specific clinical manifestations of primary cutaneous aspergillosis, a definitive diagnosis often requires culture and histopathological examination of lesional skin tissue (3). The 2021 Consensus Guidelines for the Diagnosis and Management of Invasive Aspergillosis recommend voriconazole, combined with therapeutic drug monitoring, as the first-line treatment regimen (4).

The core of PG treatment is to inhibit the excessive immune-inflammatory response of the body through the use of glucocorticoids to control lesion progression and promote wound healing. However, the use of glucocorticoids significantly impairs immune function, resulting in decreased fungal clearance capacity and even systemic disseminated fungal infections. If anti-inflammatory treatment is postponed, the ulcer will expand due to persistent inflammatory responses, further increasing the risk of fungal colonization, forming a closed loop of “inflammation exacerbates ulcers—ulcers promote infections—infections hinder anti-inflammation.”

Therefore, in the treatment of this case, the adjustment of glucocorticoid dosage and the synergistic use of antifungal drugs were based on the dynamic balance between “the degree of infection control” and “inflammatory progression.” Initially, tentative low-intensity anti-inflammation combined with preliminary antifungal therapy not only avoided rapid ulcer progression but also preserved the body’s ability to clear fungi. In the second stage, considering the low oral bioavailability of itraconazole (approximately 55%), the antifungal regimen was upgraded to voriconazole to ensure complete control of the infection. Voriconazole exhibits higher fungicidal activity against *Aspergillus* species and can penetrate the blood–tissue barrier to reach the deep layers of the wound. Meanwhile, a low dose of glucocorticoid was maintained, which not only resolved the insufficient antifungal intensity but also created favorable conditions for skin grafting. In the third stage, skin grafts require a favorable local microenvironment, whereas persistent inflammation due to pyoderma gangrenosum (PG) may lead to graft rejection or necrosis. Therefore, we increased the anti-inflammatory intensity by approximately 50% while consolidating antifungal therapy, a strategy that can better manage potential acute inflammatory fluctuations after skin grafting and prevent graft necrosis caused by the isomorphic response or infection.

For PG patients with combined infections, while sufficient glucocorticoids are used to control the condition, an appropriate timing should be selected for surgical debridement, skin grafting, or NPWT (5). The current optimal surgical method is skin grafting combined with NPWT. After effective pretreatment of the wound with NPWT, the use of NPWT to fix split-thickness skin grafts can significantly improve the graft survival rate, reduce the number of repeated skin grafting procedures, and promote rapid intrinsic healing and good epithelialization of the wound (6, 7).

After previous treatment, the patient in this case had generally met the surgical conditions of “infection control, inflammation stabilization, and wound optimization.” At this point, skin grafting was performed to actively close the wound, break the risk chain of unhealed wounds and infections, and create room for subsequent adjustments to the immunosuppressive regimen. Postoperatively, fixation of the grafted skin with NPWT not only continuously drains wound exudate and fungal contaminants to prevent the formation of a “nutrient-rich environment” but also promotes local blood perfusion of the wound, increases tissue oxygen partial pressure, and inhibits the germination of *Aspergillus flavus* hyphae. Additionally, it can modulate the levels of inflammatory factors: upregulating IL-8, downregulating TNF-α and IL-1β, and increasing the anti-inflammatory factor IL-10. This modulation alters the inflammatory microenvironment, which may thereby block the inflammatory process and indirectly reduce the invasive ability of fungi (8, 9).

After the operation, the patient did not experience an isomorphic reaction or aggravated infection in either the original lesion or the skin-harvesting site. This case suggests a feasible clinical strategy for treating patients with cutaneous fungal infection and possible pyoderma gangrenosum. During the treatment process, it is necessary to closely monitor and control infection and appropriately balance the relationship between immunosuppressive therapy and anti-infective therapy.

## Data Availability

The original contributions presented in the study are included in the article/supplementary material, further inquiries can be directed to the corresponding author.
